# Risk factors of sitting-induced tachycardia syndrome in children and adolescents

**DOI:** 10.1371/journal.pone.0265364

**Published:** 2022-03-18

**Authors:** Yuanyuan Wang, Zhenhui Han, Yaru Wang, Yongqiang Yan, Zhitao Pan, Hanwen Zhu, Hongxia Li, Chunyan Tao, Ping Liu, Yuli Wang, Chaoshu Tang, Hongfang Jin, Junbao Du

**Affiliations:** 1 Department of Pediatrics, Peking University First Hospital, Beijing, China; 2 Department of Cardiology, Children’s Hospital of Kaifeng, Henan, China; 3 Department of Pediatric Surgery, Children’s Hospital of Kaifeng, Henan, China; 4 Key Laboratory of Molecular Cardiovascular Sciences, Ministry of Education, Beijing, China; 5 Department of Physiology and Pathophysiology, Health Science Centre, Peking University, Beijing, China; Karnali Academy of Health Sciences, NEPAL

## Abstract

**Background:**

The study was designed to explore the risk factors for sitting-induced tachycardia syndrome (STS) in children and adolescents.

**Methods and results:**

In this case-control study, 46 children with STS and 184 healthy children and adolescents were recruited. Demographic characteristics, lifestyle habits, allergy history, and family history were investigated using a questionnaire. The changes in heart rate and blood pressure from supine to sitting were monitored using a sitting test. The possible differences between STS patients and healthy children were analyzed using univariate analysis. Logistic regression analysis was used to explore the independent risk factors for STS. Univariate analysis showed that the daily sleeping time of the STS children were significantly shorter than that of the control group [(8.8 ± 1.2) hours/day vs. (9.3 ± 1.0) hours/day, P = 0.009], and the proportion of positive family history of syncope in the STS patients was higher than the controls (4/42 vs. 3/181, P = 0.044). Multivariate logistic regression studies showed that reduced daily sleeping time was an independent risk factor of STS in children (P = 0.006). Furthermore, when daily sleeping time was prolonged by 1 h, the risk of STS was decreased by 37.3%.

**Conclusion:**

Reduced daily sleeping was an independent risk factor for STS in children and adolescents.

## Introduction

Sitting-induced tachycardia syndrome (STS) is a new syndrome which was firstly proposed in 2020 [1]. STS is defined as an increase in heart rate (HR) of >25 beats per minute (bpm) in the sitting position within 3 minutes compared to the supine position [1]. STS is accompanied by symptoms such as palpitations, suffocation, dizziness, headache, and even syncope, with the predisposing factors such as persistent sitting or postural change from supine to sitting [1–3]. This condition seriously affects the quality of life and learning, damages physical and mental health, and increases the economic burden on the affected individual’s family and society [4, 5]. Therefore, studying the risk factors of STS in children and adolescents and improving the prevention of such diseases are urgent issues in the field.

Previous studies have shown that in normal participants, when the body position changes from supine to sitting or standing, gravity causes a short-term decline in blood pressure (BP) due to a mild decrease in venous blood return and cardiac output. This decrease in BP activates the sympathetic nervous system and withdraws the parasympathetic nervous system to stimulate the positive inotropic cardiac effects to maintain a relatively stable circulatory state [6]. In patients with hypovolemia, insufficient venous return leads to a significant reduction in cardiac output in the sitting position, which triggers reflex tachycardia. Previous studies have also shown that water and saline intake are related to the circulating blood volume [7–9]. In short, we speculated that when children with STS changed from the supine to sitting position, insufficient fluid and saline intake and decreased circulating blood volume would result in a significant cardiac output reduction in the sitting position, thus triggering reflex tachycardia. Therefore, water intake might be related to the occurrence of STS in children. In addition, female sex hormones have the potential to reduce blood volume and vasodilator effects, and androgen treatment could significantly improve these symptoms [10]. In other words, girls were also prone to experience reflex tachycardia in the sitting position. Therefore, there might be a correlation between sex and STS occurrence.

Studies have also shown that a lack of sleep can lead to decreased physical function and increased risk of cardiovascular disease [5, 11]. Compared with a control group, the nocturnal catecholamine levels were increased in patients with insomnia [12]. Therefore, it is possible that a lack of sleep in children with STS is associated with increased levels of catecholamine in the body, which also causes tachycardia [13, 14]. In allergic diseases, the release of histamine, bradykinin, prostaglandin, and other substances can relax vascular smooth muscle and increase vascular permeability, and subsequently cause an excessive increase in HR [15, 16]. Based on these findings, we hypothesized that the risk of STS might be associated with the daily water, daily sleeping hours, and allergy history.

Therefore, this study aimed to investigate the risk factors of STS in children and adolescents by analyzing demographic characteristics, lifestyle habits, history of allergies, and family history of syncope.

## Methods

### Study design

This case-control study included pediatric STS as a case group based on the corresponding diagnostic criteria [1], and healthy children according to the history taking, physical examination and laboratory hemodynamic investigation as a control group.

### Study participants

**Fig 1:**
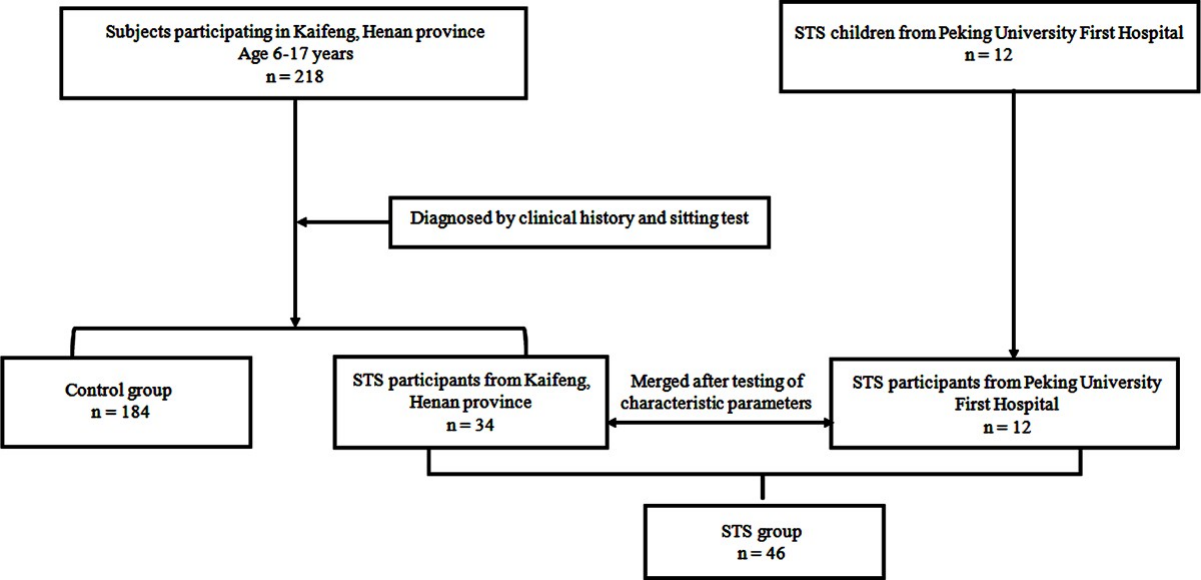
Flowchart of participant selection. STS, sitting-induced tachycardia syndrome.

In June to October 2018, we recruited 218 participants of school-aged children and adolescents (ages 6 to 17 years) from 4 primary school and middle school in Kaifeng, Henan province. Among them, 184 participants served as the healthy controls based on the history-taking, physical examination, and laboratory investigation, and the remaining 34 were diagnosed as STS by the Department of Pediatrics of Peking University First Hospital, China according to the diagnostic criteria of STS [1]. We also collected another 12 children diagnosed as STS from October 2017 to January 2019 in the Department of Pediatrics of Peking University First Hospital, China. Therefore, the final sample consisted of 230 participants, including 119 boys (51.7%), among whom 46 suffered from STS (STS group) and 184 served as the healthy controls (control group) [19 boys (41.3%) in the STS group and 100 boys (54.3%) in the control group]. A flowchart of study enrollment was shown in Fig 1.

### Sample size calculation

The sample size was roughly calculated based on the principle of logistic regression analysis. As each independent variable required at least 10 participants, at least 80 participants were needed.

### Inclusion criteria and exclusion criteria of participants

Inclusion criteria: School-aged children and adolescents who received routine physical examinations from 4 primary and middle schools in Kaifeng City, Henan Province. And school-aged children and adolescents were diagnosed as STS in the Department of Pediatrics of Peking University First Hospital from October 2017 to January 2019. The age ranges from 6 to 17 years old.

Exclusion criteria: Among them, girls who were menstruating were excluded to avoid the confounding factors. Participants were also excluded if they had symptoms including a cold, fever, vomiting, diarrhea, abdominal pain, or rash in the past 2 weeks. Drug use in the past 3 days or having organic heart disease, cerebrovascular disease, thyroid dysfunction, congenital adrenal hyperplasia, anemia, liver and kidney insufficiency, tumors, and other organic diseases were excluded.

### Diagnostic criteria of sitting-induced tachycardia syndrome

The diagnostic criteria for STS were as follows [1]: (1) predisposition, such as sudden postural change from the supine to sitting and long-term sitting position; (2) sitting-related symptoms such as dizziness, headache, chest tightness, and even fainting; (3) during a sitting test, HR increase by ≥25 bpm within 3 min from supine to sitting position compared to that in the supine position; (4) during a sitting test, no significant change in BP; (5) sitting-related symptoms caused by cardiogenic reasons, epilepsy, hypoglycemia, and other causes were excluded.

This study was approved by the Peking University First Hospital Human Research Ethics Committee (2018 [201]) and followed the Declaration of Helsinki. The verbal consent was approved by the Peking University First Hospital Human Research Ethics Committee and was obtained from the participants’ guardians.

### Questionnaire

The questionnaire referred to the reference [7] and was modified by the experts. The final questionnaire was collected by the trained investigators. The content of the questionnaire included demographic characteristics such as gender, age, address, telephone number, history of sitting syncope, sitting-related symptoms, motion sickness, family history of syncope, daily water intake, daily sleeping hours, and food or drug allergy history. This information was provided to the participants with the help of their guardians. Daily water intake referred to a graduated cup [7, 17]. Daily sleeping time was defined as the average daily sleeping time in the previous week.

### Measurement of height and weight

Height and weight were measured using the Height and Weight Tester HW-600 (Kaiyuan Electronics Co, Ltd, Zhengzhou, China). Before measurement, all participants were required to remove their shoes, socks, and hats, and avoid exercise and urination before recording their weight accurately to the closest 0.1 kg. The participant was required to stand upright on the weighing platform, with their back leaning on the height measuring rod, and their eyes straight ahead. The examiner then adjusted the measuring plate until it touched the participants’ head, and recorded the height accurately to the closest 0.1 cm. Body mass index (BMI) was calculated as weight divided by height squared (kg/m^2^).

### Sitting test

A Dash 2000 multi-channel ECG monitor (General Electric, Schenectady, New York, USA) was used for the sitting test. The specific process was as follows: the participant was required to lie quietly in a supine position for 5–10 minutes until the HR and BP were stable, and the HR and BP were recorded in a supine position at this time. Then, the participant was required to take a sitting position, while keeping the thighs in a horizontal position, the knee joints near 90°, and upper limbs hang down naturally on both sides of the body for 10 minutes. During this period, any abnormal discomfort and changes in HR and BP were recorded for each minute. If the participant could not tolerate the examination, the test was stopped, and the participant was allowed to rest in a supine position until recovery.

### Statistical analysis

IBM SPSS Statistics software (version 23.0) was used for data analysis. The Shapiro-Wilk test was used to assess data distribution normality, and the measurement data are expressed as the mean ± standard deviation. When the two groups were in the normal distribution, the independent t-test was used to compare the groups; otherwise, the Mann-Whitney U test was used in the univariate analysis. The enumeration data are expressed as the number of cases (n), and the chi-square test (χ^2^ test) was used to compare differences between groups. Confounding factors were controlled since the comparison of variables showed no significant differences among STS patients from different sources. Logistic regression analysis (conditional-forward method) was used to determine the risk factors for STS, and the odds ratio was calculated. Statistical significance was set at P < 0.05.

## Results

### Comparison of demographic characteristics, lifestyle habits, allergy history, and family history of syncope between groups

In the STS group, 34 children with STS from Kaifeng city [15 boys (44.1%), at an average age of (11.1 ± 2.2) years] and 12 children with STS from Peking University First Hospital [4 boys (33.3%), at average age (12.2 ± 1.9) years] were recruited. There were no statistical difference in demographic characteristics [gender (χ^2^ = 0.097), age (t = -1.491) and BMI (Z = -0.400)], living habits [daily water intake (Z = -1.371), daily sleep time (Z = -1.609), and history of motion sickness (χ^2^ = 0.474)], history of food and drug allergy (χ^2^ = 0.045) and syncope family history (χ^2^ = 0.296) (all P > 0.05) between the 2 groups, suggesting that the children with STS from the two regions could be combined into one group (STS group) for further statistical analysis (Table 1). In the study, 184 healthy children were in the control group, including 100 boys (54.3%), at an average age of 11.0 ± 2.1 years old.

**Table 1:** Distribution of characteristics of children with STS from different sources.

Groups	Cases, n	Gender (M/F)	Age, yrs	BMI, kg/m^2^	Daily water intake, ml/d	Daily sleeping hours, h/d	Family history of syncope (yes/no)	Motion sickness history (yes/no)	Allergy history (yes/no)
K-STS	34	15/19	11.1 ± 2.2	18.2 ± 2.6 *	628 ± 325 *	9.0 ± 1.0 *	2/32	14/20	3/31
P-STS	12	4/8	12.2 ± 1.9	19.2 ± 3.4	796 ± 347	8.2 ± 1.6	2/10	7/5	2/10
χ^2^/Z/t	-	0.097	-1.491	-0.400	-1.371	-1.609	0.296	0.474	0.045
P value	-	0.756	0.143	0.689	0.170	0.108	0.586	0.491	0.833

STS, sitting-induced postural tachycardia syndrome; K-STS, children, and adolescents with sitting tachycardia syndrome from Kaifeng city; P-STS, children and adolescents with sitting tachycardia syndrome from Peking University First Hospital; BMI, body mass index; kg/m^2^, kilogram per square meter. Date is shown as mean ± SD or numbers. *Non-normal distribution.

Table 2 shows the comparison of characteristics between the STS and control groups. There were no significant differences in gender (χ^2^ = 2.507), age (Z = -1.024), BMI (Z = -1.256), daily water intake (Ζ = -0.345), motion sickness history (χ^2^ = 1.173), and food or drug allergy history (χ^2^ = 1.016) between the STS and control groups (all P > 0.05). While compared with that of the control group, the daily sleeping time was significantly short in the STS group (Z = -2.627, P = 0.009) and the proportion of positive family history of syncope was high in the STS group (χ^2^ = 4.061, P = 0.044).

**Table 2:** Comparison of characteristics between STS group and control group in children.

Groups	Cases, n	Gender (M/F)	Age, yrs	BMI, kg/m^2^	Daily water intake, ml/d	Daily sleeping hours, h/d	Family history of syncope (yes/no)	Motion sickness history (yes/no)	Allergy history (yes/no)
Control	184	100/84	11.0 ± 2.1 *	18.0 ± 3.1 *	672 ± 369 *	9.3 ± 1.0 *	3/181	68/116	12/172
STS	46	19/27	11.4 ± 2.1 *	18.5 ± 2.8 *	672 ± 335 *	8.8 ± 1.2 *	4/42	21/25	5/41
χ^2^/Z	-	2.507	-1.024	-1.256	-0.345	-2.627	4.061	1.173	1.016
P value	-	0.113	0.306	0.209	0.730	0.009	0.044	0.279	0.313

STS, sitting-induced postural tachycardia syndrome; BMI, body mass index; kg/m^2^, kilogram per square meter.

Date is shown as mean ± SD or numbers. * Non-normal distribution.

### Analysis of risk factors in children with sitting-induced tachycardia syndrome

The variables with statistical differences (P < 0.05) in the univariate analysis (daily sleep time and positive family history of syncope) were included in the multivariate analysis. There was no collinearity between the 2 parameters before being introduced into the multivariate analysis. Logistic analysis revealed that the reduced daily sleeping time was an independent risk factor for STS in children (P = 0.006). When the daily sleeping time prolonged by 1 h, the risk of STS would be decreased by 37.3% (Table 3).

**Table 3:** Logistic multivariate regression analysis of variables.

Characteristics	B	SE	Wald	P value	OR (95%CI)
Family history of syncope (yes/no)	1.528	0.796	3.685	0.055	4.610 (0.968–21.948)
Daily sleeping hours	-0.467	0.171	7.442	0.006	0.627 (0.448–0.877)
Constant	2.768	1.536	3.248	0.072	-

SE, standard error; OR, odds ratio; CI, confidence interval. Characteristic enrolled in the logistic multivariate regression analysis: daily sleeping hours and family history of syncope.

The logistic equation was Log (P) = 2.768–0.467×X_1_, where X_1_ was defined as daily sleeping hours.

## Discussion

The present study revealed that the daily sleeping time was significantly associated with the occurrence of pediatric STS. Specifically, the risk of pediatric STS would be decreased by 37.3% when the daily sleeping time prolonged by 1 h. Therefore, appropriately increasing the daily sleeping time could reduce the risk of STS.

To date, the mechanism for the correlation between daily sleeping duration and the occurrence of STS has been unclear. Based on current research evidence [12, 18–20], the imbalance of autonomic nervous function and endocrine hormones caused by the sleep deficiency might be related to the occurrence of pediatric STS. A study reported that the level of nocturnal catecholamines in patients with insomnia was higher than that in the control group [12]. Follenius’ s study showed that insufficient sleep or sleep interruption was associated with increased cortisol levels in plasma [18]. Furthermore, sleep deprivation as a typical stress can not only activate the hypothalamus-pituitary-adrenal cortex axis leading the obvious increase of serum cortical and the locus ceruleus-norepinephrine/sympathetic-adrenal medulla axis resulting in the obvious increase in serum dopamine, epinephrine and norepinephrine, but also activate the rennin-angiotensin system leading an remarkable increase in serum rennin and angiogenesis II [19]. Animal studies suggested that after 48 h of sleeping deprivation, the sympathetic nerve activity of rats was significantly excited and vagal nerve activity was inhibited, indicating an autonomic nerve imbalance [20]. In addition, sleeping disorder was common in pediatric postural tachycardia syndrome (POTS) that was thought to have similar hemodynamic characteristics to pediatric STS [1, 21]. Sleeping duration of less than 8 hours was deemed to be a risk factor for pediatric POTS [7]. Currently, sleeping intervention therapy has been proven to be an effective treatment for POTS symptoms [22], and another study showed that salivary cortisol concentration when awake could effectively predict the efficacy of sleep promotion therapy for pediatric POTS [23]. Therefore, it can be speculated that the reduced sleeping duration increased sympathetic excitement, causing endocrine hormone secretion disorders. In other words, children with insomnia may lead to a maladaptive daytime sympathetic excitement, which might be the cause of STS [24]. When the position of STS children with reduced sleeping changes from a supine to sitting position, the cardiac blood volume return would be obviously insufficient, the reflex would cause excessive vagal nerve truncation and excessive enhancement of sympathetic nerve activity in children, which might lead to STS and increase the volume of cardiac blood to ensure the adequate cerebral blood supply.

As such, reduced sleeping time was the risk factor for pediatric STS. The findings shed light on our understanding of the risk factor of this disease. At the same time, the study would provide useful advice for children and their guardians to avoid the risk factor to strengthen the prevention and treatment of this disease in the future.

## Strengths and limitations

However, the study still had some limitations. First, the sample size was still relatively small, and a larger-scale investigation is needed in the future. Furthermore, although the study participants were from the central and northern China, the lifestyle habits and demographic characteristics of the participants might not fully represent the whole population. At last, external validation is lacking as the pilot nature of the present study. Even though, the significance of this research cannot be ignored. It is the first time to determine the risk factor of a newly discovered disease STS in children and adolescents. The findings would provide the necessary knowledge and information to the further research and prevention of this condition.

## Conclusion

Collectively, the data showed that reduced daily sleeping time was the independent risk factor for STS in children and adolescents. However, the pathogenesis of the disease has not yet been fully understood and more studies are still needed in the future.
